# Modeling language and cognition with deep unsupervised learning: a tutorial overview

**DOI:** 10.3389/fpsyg.2013.00515

**Published:** 2013-08-20

**Authors:** Marco Zorzi, Alberto Testolin, Ivilin P. Stoianov

**Affiliations:** ^1^Computational Cognitive Neuroscience Lab, Department of General Psychology, University of PadovaPadova, Italy; ^2^IRCCS San Camillo Neurorehabilitation HospitalVenice-Lido, Italy; ^3^Institute of Cognitive Sciences and Technologies, National Research CouncilRome, Italy

**Keywords:** neural networks, connectionist modeling, deep learning, hierarchical generative models, unsupervised learning, visual word recognition

## Abstract

Deep unsupervised learning in stochastic recurrent neural networks with many layers of hidden units is a recent breakthrough in neural computation research. These networks build a hierarchy of progressively more complex distributed representations of the sensory data by fitting a hierarchical generative model. In this article we discuss the theoretical foundations of this approach and we review key issues related to training, testing and analysis of deep networks for modeling language and cognitive processing. The classic letter and word perception problem of McClelland and Rumelhart (1981) is used as a tutorial example to illustrate how structured and abstract representations may emerge from deep generative learning. We argue that the focus on deep architectures and generative (rather than discriminative) learning represents a crucial step forward for the connectionist modeling enterprise, because it offers a more plausible model of cortical learning as well as a way to bridge the gap between emergentist connectionist models and structured Bayesian models of cognition.

## Introduction

A fundamental issue in the study of human cognition is what computations are carried out by the brain to implement cognitive processes. The connectionist framework assumes that cognitive processes are implemented in terms of complex, non-linear interactions among a large number of simple, neuron-like processing units that form a neural network (Rumelhart and McClelland, 1986). This approach has been used in cognitive psychology—often with success—to develop functional models that clearly represent a great advance over previous verbal-diagrammatic models because they can produce simulations of learning, skilled performance, and breakdowns of processing after brain damage. One paradigmatic example is the connectionist modeling of visual word recognition and reading aloud, which has often provided key theoretical and methodological advances with broad influences well-beyond the language domain (e.g., McClelland and Rumelhart, 1981; Seidenberg and McClelland, 1989; Plaut and Shallice, 1993; Plaut et al., 1996). Connectionist models of the reading processes can produce highly detailed simulations of human performance, accounting for a wide range of empirical data that include reaction times and accuracy of skilled readers at the level of individual words, the development of reading skills in children, and the impaired performance of dyslexic individuals (Plaut et al., 1996; Zorzi et al., 1998; Harm and Seidenberg, 1999, 2004; Perry et al., 2007, 2010, 2013). Despite significant progress in the attempt to improve the architectural and learning principles incorporated in neural network models (see O'Reilly, 1998; O'Reilly and Munakata, 2000), much modeling work in psychology is still based on the classic neural network with one layer of hidden units (i.e., a “shallow” architecture) and error backpropagation (Rumelhart et al., 1986) as learning algorithm—a choice that is typically seen as a compromise to achieve efficient learning of complex cognitive tasks. We argue below that a key step forward for connectionist modeling is the use of networks with a “deep” architecture (Hinton, 2007, 2013) and where most of the learning is generative rather than discriminative (Box 1).

Box 1 **Glossary.**

**Boltzmann machine**

Stochastic neural network of symmetrically connected, neuron-like units whose dynamics is governed by an energy function. The input to the network is given through a layer of visible units, while another layer of hidden units is used to model the latent causes of the data. A variant known as Restricted Boltzmann Machine (RBM) is obtained by removing within-layer lateral connections to form a bipartite graph, allowing to perform efficient inference and learning.

**Contrastive divergence**

Objective function that allows to efficiently train RBMs by approximating the log-likelihood gradient, without requiring to run a Markov chain to convergence.

**Deep belief network**

Hierarchical generative model composed of a stack of RBMs, which can be greedily trained layer-wise in an unsupervised fashion. The whole network can be eventually fine-tuned with supervised learning to perform discriminative tasks.

**Deep learning**

Machine learning framework that exploits multiple layers of hidden units to build hierarchical internal representations of the input data.

**Discriminative learning**

Learning approach whose objective is to map the observed variables *X* into corresponding output variables *Y*, usually by modeling the conditional distribution *P*(*Y*|*X*), optimizing classification boundaries, or by approximating a function *Y* = *f*(*X*). This approach requires labeled examples (i.e, a teaching signal for supervised learning).

**Generative learning**

Learning approach whose objective is to model the joint distribution *P*(*X, Y*) of observed and latent variables, typically using a likelihood-based criterion. This approach does not require labeled data (i.e., learning is unsupervised).

**Graphical models**

Probabilistic models in which the topology of a graph defines conditional independecies between random variables, allowing to efficiently represent complex joint distributions through factorization.

The shallow architecture of the prototypical multi-layer neural network (Rumelhart et al., 1986) does not capture the hierarchical organization of the cerebral cortex. Hierarchical processing is thought to be a fundamental characteristic of cortical computation (Hinton, 2007; Clark, 2013) and it is a key feature of biologically inspired computational models of vision (Riesenhuber and Poggio, 1999). The idea of a deep network with a hierarchy of increasingly complex feature detectors can be traced back to the Interactive Activation Model (IAM) of letter and word perception (McClelland and Rumelhart, 1981), but this seminal proposal did not transfer to connectionist learning models because the error backpropagation algorithm had little success in training networks with many hidden layers (Hinton, 2007, 2013). Another key assumption of the IAM that did not readily transfer to connectionist learning models is the mixing of bottom–up and top–down processing through recurrent feedback. Finally, the widespread use of the error backpropagation algorithm in connectionist modeling, leaving aside its lack of biological plausibility (O'Reilly, 1998), implies subscription to the dubious assumption that learning is largely discriminative (e.g., classification or function learning) and that an external teaching signal is available at each learning event (that is, all training data is labeled). This learning regimen is exceptional in the real world. Reinforcement learning (Sutton and Barto, 1998) is a plausible alternative, but there is a broad range of situations where learning is fully unsupervised and its only objective is that of building rich internal representations of the sensory world (Hinton and Sejnowski, 1999). Notably, the learned internal model can then be used to infer causes and make predictions (Dayan et al., 1995; Hinton and Ghahramani, 1997; Friston, 2005; Hinton, 2010b; Huang and Rao, 2011; Clark, 2013).

Unsupervised learning has a long history, but the classic learning algorithms have important limitations. Some develop a representation that is distributed but also linear (Oja, 1982), which implies that higher-order information remains invisible. Others develop a representation that is non-linear but also localist, that is one in which each observation is associated to a single hidden unit (Rumelhart and Zipser, 1985; Kohonen, 1990). For these reasons, their application to modeling complex cognitive functions has been limited. An important breakthrough in unsupervised learning is the use of statistical principles such as maximum likelihood and Bayesian estimation to develop generative models that discover representations that are both distributed and non-linearly related to the input data (Hinton and Ghahramani, 1997). A generative model is a probabilistic model that captures the hidden (latent) causes of the data, thereby providing a sensible objective function for unsupervised learning. In other words, the “learner” estimates a model, without any supervision or reward, that represents the probability distribution of the data. Generative models are appealing because they make strong suggestions about the role of feedback connections in the cortex and are consistent with neurobiological theories that emphasize the mixing of bottom–up and top–down interactions in the brain: bottom–up inputs convey sensory information, whereas internal representations form a generative model that predicts the sensory input via top–down activation (Hinton and Ghahramani, 1997). Learning can be viewed as maximizing the likelihood of the observed data under the generative model, which is equivalent to discovering efficient ways of coding the sensory data (Ghahramani et al., 1999). Notably, the application of these algorithms to natural images has been shown to generate receptive field properties similar to those observed in the visual cortex (Rao and Ballard, 1999).

Generative learning can be implemented in the framework of recurrent stochastic neural networks with hidden units (Hinton, 2002). However, one hidden layer can be insufficient for modeling structured and high-dimensional sensory data. In contrast, a network with many hidden layers, that is a deep network, can learn a more powerful *hierarchical generative model* (Hinton and Salakhutdinov, 2006; Hinton et al., 2006). Note that a good generative model of the data can be a very useful starting point for later discriminative learning (Hinton, 2007; Stoianov and Zorzi, 2012). The internal representations obtained from generative learning can be the input to a variety of classification or function learning tasks, thereby exploiting re-use of learned features (Bengio et al., 2012). Moreover, the internal model might be refined through supervised learning to strengthen the features that are most informative for solving a specific classification task (Hinton and Salakhutdinov, 2006; also see Love et al., 2004, for a related modeling approach to category learning). Indeed, it has been shown that human category learning implies flexibility in the use and creation of perceptual features (Schyns et al., 1998) and that different types of features might be extracted according to the nature of the learning task (e.g., unsupervised vs. supervised; Love, 2002).

The goal of the present article is to provide a tutorial overview of generative learning in deep neural networks to highlight its appeal for modeling language and cognition. We start with a brief review of the theoretical foundations of generative learning and deep networks. We then discuss various practical aspects related to training, testing and analyzing deep networks, using the classic letter and word perception problem of McClelland and Rumelhart (1981) as a tutorial example. The emergence of a hierarchy of orthographic representations through deep unsupervised learning is particularly interesting (also see Di Bono and Zorzi, under review) because it can revisit the hard-wired architecture of the IAM. The idea that perception of written words involves the sensitivity to increasingly larger orthographic units is also supported by recent neuroimaging findings (Dehaene et al., 2005; Vinckier et al., 2007).

## Learning a generative model: restricted boltzmann machines

Here we consider a class of neural networks known as Boltzmann Machines (hereafter BM; Ackley et al., 1985). These are stochastic associative networks that observe and model data by using local signals only. BMs can be interpreted as undirected graphical models (Jordan and Sejnowski, 2001; see Box 2) where learning corresponds to fitting a generative model to the data. Despite the appeal of BMs as plausible models of cortical learning, their use was strongly discouraged by the very high computational demand of the original learning algorithm, until the recent development of *contrastive divergence (CD) learning* (Hinton, 2002). CD makes learning of BMs practical, even for large networks (see below).

Box 2 **Probabilistic Graphical Models.**

The framework of probabilistic graphical models (Koller and Friedman, 2009) provides a general approach to model arbitrarily complex statistical distributions, which can involve a large number of stochastic variables that interact together. Graphical models allow us to describe complex relations between variables by exploiting the *structure* of their joint distribution, since in general their interactions are not globally defined but instead each variable is only influenced by a limited subset of “neighbors.” The topology of a graphical model explicitly defines the scope of interaction of each variable (represented by a node in the graph) by highlighting the set of independecies that hold in the distribution. This allows to *factorize* a joint probability distribution using local conditional probabilities.

**Figure 1:**
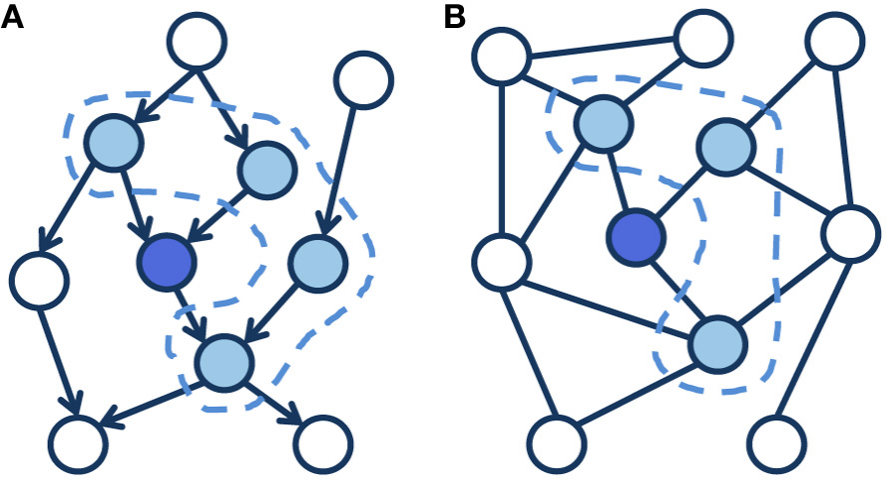
**(A)** A directed graphical model, also known as Bayesian network. **(B)** An undirected graphical model, also known as Markov network. In both graphs, the dashed line highlights the Markov blanket of the blue node.

Graphical models can have *directed* connections between variables, such as in Bayesian networks (Figure 1A), or *undirected* connections, such as in Markov networks (Figure 1B). Both types of connections might be present in the same graph, thus forming a *hybrid* model. Although they share the same underlying theoretical framework, Bayesian and Markov networks have rather different representational and computational characteristics. In directed models, the semantic of connections defines a “parent of” relationships between linked variables, while in undirected models the connections are symmetric and therefore only encode a sort of “degree of affinity” between linked variables. This leads to a different representation of independencies between nodes of the graph: in Bayesian networks, each node is conditionally independent from all the others given its parents, its children and the parents of its children, while in undirected models each node is conditionally independent from all the others given the nodes directly connected to it [i.e., its “Markov blanket” (Pearl, 1988), highlighted in Figure 1]. In both cases, these conditional independencies can be exploited to derive efficient inference and learning procedures even in the presence of a large number of variables, because only the Markov blanket of a certain node is required in order to sample from its conditional distribution.

In the case of undirected graphical models, each edge is associated with a certain function, known as *factor*, which takes as input the values of the nodes connected by the edge and gives as output a scalar value that represents the affinity between them: a high value indicates that the two variables are likely to be strongly related, while a low value indicates a weak relation. The joint distribution of all the variables in the graph can be efficiently defined as a product of such local factors:

$$P(X_{1},X_{2},\ldots ,X_{n})=\frac{1}{Z}\prod_{i}\phi_{i}(D_{i})$$

where *D*_*i*_ represents the scope of each factor ϕ_*i*_ (i.e., which variables it involves) and *Z* is a global normalization constant called *partition function*, which ensures dealing with legal probabilities summing up to 1.

BMs consist of a set of stochastic units, fully connected with symmetric weights and without self-connections, where each unit fires with a probability depending on the weighted sum of its inputs. Data patterns are represented by the activation of “visible” units. An additional layer of “hidden” units captures high-order statistics and represent the latent causes of the data. Inspired by statistical mechanics, the model behavior is driven by an energy function *E* that describes which configurations of the units are more likely to occur by assigning them a certain probability value:

$$p(v,\;h)=\frac{e^{-E(v,\;h)}}{Z}$$

where *v* and *h* are, respectively, the visible and hidden units and *Z* is a normalizing factor known as *partition function*, which ensures that the values of *p* constitute a legal probability distribution (i.e., summing up to 1). The network state changes in a way that allows the gradual decrease of the associated energy, modulated by a “temperature” parameter *T* so that at higher temperatures an occasional increase of energy is also permitted to avoid local minima. To achieve local energy minimum (equilibrium), *T* is gradually decreased (*simulated annealing)*. The learning procedure minimizes the Kullback-Liebler divergence between the data distribution and the model distribution. Accordingly, for each pattern the network performs a data-driven, *positive phase* (+) and a model-driven, *negative phas*e (–). In the positive phase the visible units are clamped to the current pattern and the hidden layer settles to a stable activation state. In the negative phase all units are unclamped and the network is run [using a Markov Chain Monte Carlo (MCMC) algorithm; see Box 3] until it settles on a stable activation state over visible and hidden units, which reflects the model beliefs. After each phase, correlations between the activations of each pair of connected units are collected and used to update the network weights. Note that learning is unsupervised (i.e., the network does not learn an input–output mapping like typical multilayer networks trained with error backpropagation) and it uses only local signals and Hebbian rules. A similar form of contrastive Hebbian learning is also used in the generalized recirculation algorithm and in Leabra (O'Reilly, 1998, 2001). Learning the connection weights in the original BM is based on a maximum likelihood learning rule that is very simple and locally optimal, but unfortunately the learning algorithm is also very slow because it implies running a Markov chain until convergence (which may require an exponential time).

Box 3 **Block Gibbs sampling in RBMs.**

In a probabilistic graphical model, we are often interested in generating samples from the model distribution. A general-purpose, powerful method is the Gibbs sampling algorithm, which generates a sequence of observations that progressively approximate a specified multivariate probability distribution (Geman and Geman, 1984). Gibbs sampling belongs to the family of MCMC methods, which draw samples from a probability distribution by constructing a Markov chain that has the desired distribution as its equilibrium distribution (Andrieu et al., 2003). Under certain conditions, after an initial *burn-in* phase the Markov chain will converge to the stable distribution. The basic idea of Gibbs sampling is to construct the Markov chain so that one particular variable is sampled at each step *given* the current values of *all the other variables*. After repeating this process iteratively for enough time, the chain will generate samples from the target joint distribution. Notably, Gibbs sampling can exploit the structure of the graph (i.e., the conditional independecies between variables) to speed up this process: since the value of each node is only influenced by its Markov blanket (see Box 2), if two variables are conditionally independent given the current evidence (i.e., their Markov blanket is observed) they can be sampled at the same time. This variant of the algorithm is known as *block Gibbs sampling*.

**Figure 2:**
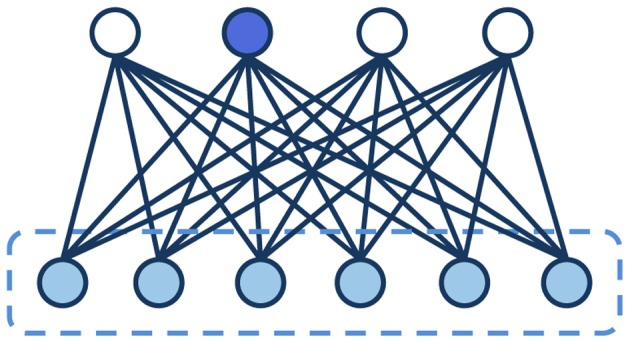
**Graphical representation of a Restricted Boltzmann Machine.** The dashed line highlights the Markov blanket of the blue hidden unit, which corresponds to the whole layer of visible units.

In the case of Boltzmann Machines, learning requires sampling from the joint distribution of visible and hidden variables in order to compute visible-hidden correlations on the model expectations. If the connectivity of the network is restricted, as in the RBM, the sampling process can be significantly speeded up by using block Gibbs sampling. Indeed, the units of the same layer become conditionally independent if there are no intra-layer connections; that is, in RBMs the Markov blanket of a hidden unit corresponds to the visible layer, and vice versa (Figure 2). This allows to sample all units of the same layer in parallel.

The breakthrough that led to CD learning (Hinton, 2002; also see Welling and Hinton, 2002; for a mean field version) is the finding that the negative phase does not need to be run until equilibrium (i.e., full convergence). If sampling starts from the hidden unit state computed in the positive phase (i.e., driven by the data), correlations computed after a fixed number of steps in the Markov chain are sufficient to drive the weights toward a state in which the input data will be accurately reconstructed. Hence, CD learning approximates the gradient of the log-likelihood of the learning data by performing only few iterations, which in practice gives good results even with a single step (*CD-1*). After computing the model's reconstruction, weights are updated by contrasting visible-hidden correlations computed on the data vector (*v*^+^*h*^+^) with visible-hidden correlations computed on the reconstruction (*v*^−^*h*^−^):

$$\Delta W=\eta (v^{+}h^{+}-v^{-}h^{-})$$

where η is the learning rate. Importantly, a restriction to the architecture of the BM by not allowing intra-layer connections (RBM; Hinton, 2002) makes learning extremely fast. The energy function for RBMs is defined as:

$$E(v,h)=-b^{T}v-c^{T}h-h^{T}Wv$$

where *W* is the matrix of connections weights and *b* and *c* are the biases of visible and hidden units, respectively. In RBMs, the update of units in one layer no longer requires any iterative settling because they are conditionally independent given the state of the other layer. That is, the sampling process is speeded up by performing block Gibbs sampling (see Box 3) over visible and hidden units (i.e., all units in a layer are sampled in a single step).

Examples of application of CD learning in connectionist modeling studies include numerical cognition (Stoianov et al., 2002, 2004; Zorzi et al., 2005) and space coding for sensorimotor transformations (De Filippo De Grazia et al., 2012).

## Learning a hierarchical generative model: deep belief networks

**Figure 3:**
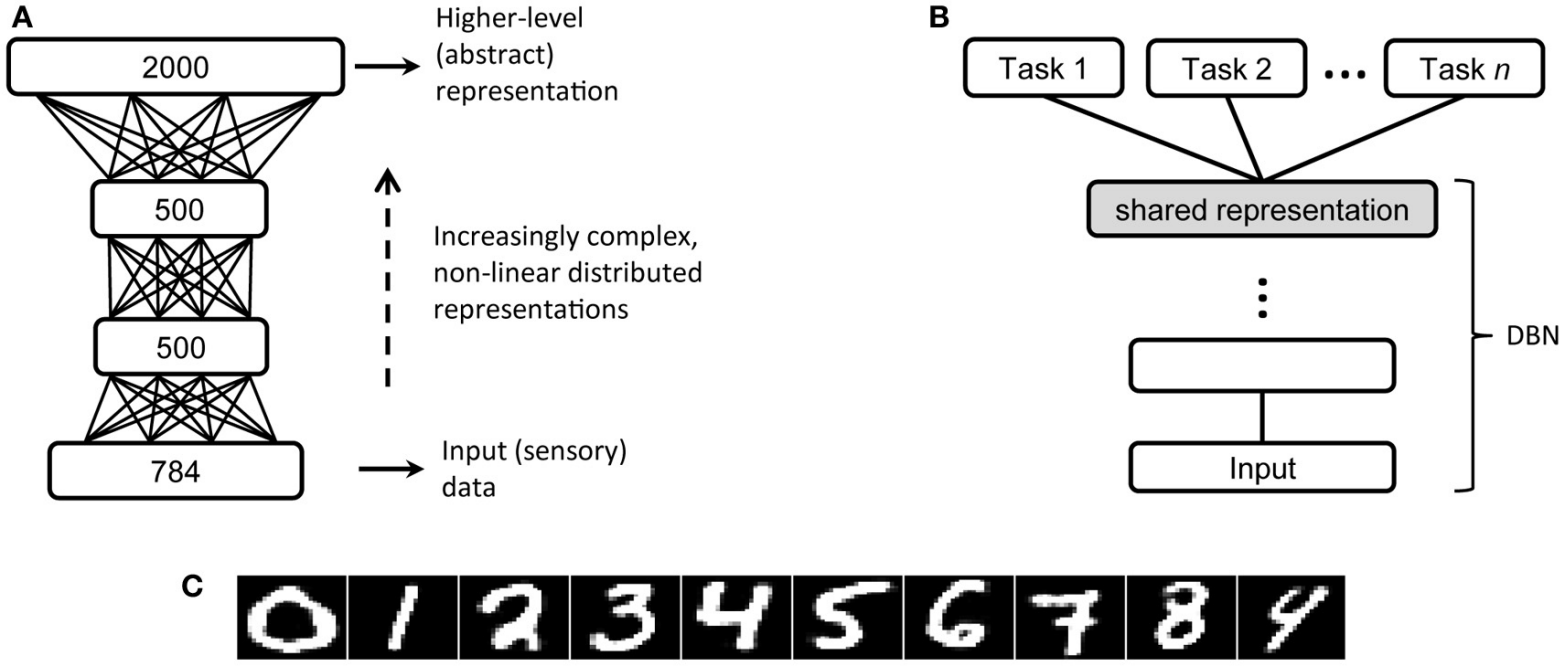
**(A)** Architecture of the DBN with three hidden layers used in the MNIST handwritten digit recognition problem (Hinton and Salakhutdinov, 2006). **(B)** A typical transfer learning scenario, on which high-level, abstract representations are first extracted by deep unsupervised learning and then used to perform a variety of supervised tasks [adapted from Bengio et al. (2012)]. **(C)** Reconstructions of MNIST digit images made by the deep network.

RBMs can be used as building blocks of more complex architectures, where the hidden variables of the generative model can be organized into layers of a hierarchy (Figure 3A). The resulting architecture is referred to as a “deep network.” In particular, the Deep Belief Network (DBN; Hinton and Salakhutdinov, 2006; Hinton et al., 2006) is a stack of RBMs that can be trained layer by layer in a greedy, unsupervised way. The main intuition behind deep learning is that, by training a generative model at level *l* using as input the hidden causes discovered at level *l–1*, the network will progressively build more structured and abstract representations of the input data. Importantly, architectures with multiple processing levels permit an efficient encoding of information by exploiting re-use of features among different layers: simple features extracted at lower levels can be successively combined to create more complex features, which will eventually unravel the main causal factors underlying the data distribution. Indeed, it has been shown that functions that can be compactly represented by a depth *k* architecture might require an exponential number of computational elements to be represented by a depth *k–1* architecture (Bengio, 2009). Moreover, adding a new layer to the architecture increases a lower bound on the log-likelihood of the generative model (Hinton et al., 2006), thus improving the overall capacity of the network. After learning of all layers, the deep architecture can be used as a generative model by reproducing the data when sampling from the model, that is by feeding the activations of the deepest layer all the way back to the input layer. Note that the hierarchical structure of the internal representations is an emergent property of the learning algorithm. In contrast, hierarchy in classic connectionist models is typically built in by stipulating the representations to be used at more than one layer (e.g., Rumelhart and Todd, 1993; Perry et al., 2013); indeed, training of deep multi-layer perceptrons using error backpropagation is very difficult because the error gradient tends to vanish when propagated backwards through more than one hidden layer (see Hinton, 2013, for further discussion).

An important advantage of deep unsupervised learning is that the internal representations discovered by the network are not tied to a particular discriminative task, because the objective of learning is only to model the hidden causes of the data. However, once the system has developed expressive abstract representations, possible supervised tasks can be carried out by introducing additional modules, which directly operate on such high-level representations of the data and can therefore yield excellent performance in classification or function learning (Figure 3B). For example, on a popular handwritten digit recognition problem (MNIST dataset; LeCun et al., 1998), high discriminative accuracy can be obtained even by a linear classifier applied on the top-level internal representations of a DBN that was only trained to reconstruct the digit images (Testolin et al., 2013; examples of digits reconstructed by the network are reported in Figure 3C). Within this perspective, the use of an additional fine-tuning phase of the whole deep network using error backpropagation (as done in Hinton and Salakhutdinov, 2006) might be unwarranted, not only because of the biological implausibility of the learning algorithm, but also because the network would become specifically tuned to a particular task. Indeed, the idea that high-level representations obtained from (unsupervised) model learning should be usable across several tasks (Figure 3B) is referred to as “transfer learning” and it is a hot topic for the machine learning community (Bengio, 2009; Bengio et al., 2012). It is worth mentioning that machine learning researchers have recently investigated deep networks built through greedy layer-wise training of stacked autoencoders, where each autoencoder is a multilayer perceptron trained to auto-associate the input (Bengio and Lamblin, 2007; Baldi, 2012). This approach has been successful in terms of machine learning benchmarks, but it is less appealing than DBNs for cognitive modeling purposes because learning is based on error backpropagation and it is not grounded in a sound probabilistic framework. Moreover, deep autoencoders are not used as generative models to produce predictions based on top–down signals.

A final consideration concerns the computational complexity of deep learning: thanks to its efficiency, the algorithm proposed by Hinton et al. (2006) solves the problem of learning in densely connected networks that have many hidden layers. If implemented on multicore hardware, deep learning is practical even with *billions* of connections, thereby allowing the development of very-large-scale simulations (Raina et al., 2009; Dean et al., 2012; Le et al., 2012). Medium-to-large-scale simulations can even be performed on a desktop PC equipped with a low-cost graphic card (Testolin et al., 2013; see below).

## Connectionist modeling with deep networks: a tutorial

In this section we provide a practical overview on how to construct a complete DBN simulation. We illustrate how to train, test and analyze a deep network model using the classic letter and word perception problem of McClelland and Rumelhart (1981). Written word perception is particularly representative because it can be linked to one of the most influential models of language processing, McClelland and Rumehart's IAM, and more specifically to its two key assumptions: (1) a hierarchical organization of the network, with increasingly more complex levels of representation, and (2) the mixing of bottom–up and top–down processing (i.e., interactivity) to resolve ambiguity of the sensory input. Interestingly, a recent re-formulation of the IAM as a probabilistic generative model (Khaitan and McClelland, 2010) was shown to perform optimal Bayesian inference, thereby supporting the appeal of the hierarchical interactive architecture (Mirman et al., in press). A deep learning model would therefore represent an important step forward, because the hard-wired architecture of the IAM might be replaced by the hierarchical generative model learned in a DBN. In this regard, learning word perception can be seen as a stochastic inference problem where the goal is to estimate the posterior distribution over latent variables given the image of a word as input.

Though written word perception is an excellent candidate for deep learning, the complexity of the problem makes realistic simulations difficult to handle. For example, high-resolution images of whole words would require a very large network, with tens of thousands of visible units (e.g., 20,000 units for a 400 by 50 pixels image), many hidden layers and billions of connections (see Krizhevsky et al., 2012, for deep learning on a realistic object recognition problem). One possible simplification would be to split words into letter constituents and first model the perception of single letters. This might lead to sensible internal letter representations that are invariant to position, size, rotation, and noise (i.e., abstract letter identities; McClelland and Rumelhart, 1981). Alternatively, written words can be represented using small resolution images, with letters encoded as combinations of simple geometric features (the “Siple” font; McClelland and Rumelhart, 1981). We employed the latter solution for the simulations presented here.

In this tutorial we also consider deep learning of handwritten digits (MNIST database; LeCun et al., 1998) and visual numerosity estimation (Stoianov and Zorzi, 2012) in relation to the analysis of DBNs, because they represent more realistic perception problems that involve training on thousands of images. Training on a large dataset can be important for the emergence of a richer hierarchical structure of features.

### Training a DBN

As in other connectionist models, input to the network is provided as pattern of activations over visible units. Note that 2D images are vectorized; this implies that the spatial structure remains only implicit in the co-activation of neighboring visible units, but it can emerge during learning in the form of statistical regularities (see examples below). Learning a generative model does not require labeled data, that is, unlike supervised learning, each pattern does not need to possess a class label or any other form of associated target state. Nevertheless, this kind of information might still be useful for testing and analyzing the network. Note that realistic, large-scale simulations often imply abundance of unlabeled data and only a limited sample of pre-classified learning examples (see Le et al., 2012, for deep learning on millions of images randomly extracted from videos on the Internet).

A ready-to-use parallel implementation of deep unsupervised learning on graphic cards is described in Testolin et al. (2013), and it is publicly available for download^1^.

#### Network architecture

The learning algorithm tunes the parameters (i.e., weights) of a DBN with a given structure that should be specified after establishing the input domain. Here we only consider network architectures with fully connected pairs of layers (Figure 3A), but alternatives based on weights sharing like convolutional networks (LeCun et al., 1998) can simplify the learning problem by assuming identical processing applied to different portions of the image, thereby reducing the number of parameters of the model. In general, the size of a given hidden layer might be proportional to the expected number of features describing the data at a certain processing level. Intuitively, many hidden units will allow for the encoding of more specific characteristics of the data, whereas fewer units imply a greater compression of the representation and hence increase the generality of the features. A more neutral strategy with regard to the architectural choices is to keep the size of few consecutive layers constant. Finally, a large top hidden layer can be useful to unfold categories and classes, thereby facilitating linear associations to categories or other processing domains (as we will discuss in the following sections). At any rate, we advise to try several architectures, gradually increasing the number of layers and units per layer, until satisfactory results are obtained.

#### Learning tasks

**Figure 4:**
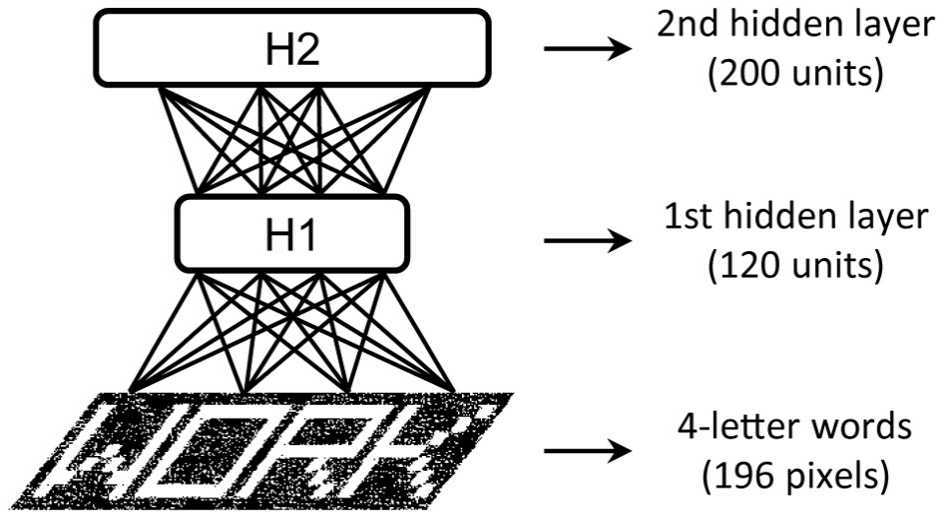
**Architecture of the DBN with two hidden layers used in the written word perception problem**.

We illustrate the tutorial with examples of increasing complexity. The first toy example is the visual perception of single letters with input consisting of black and white (b/w) images of size 7 × 7 pixels (i.e., patterns over 49 visible units). The dataset contains the images of 26 capital letters created with the schematic “Siple” font, composed of 14 basic visual features (Rumelhart and Siple, 1974). We found that a small two-layer DBN network with as few as 10 units in the first layer and 30 units in the second layer was sufficient to discover the underlying visual features. The second example extends the problem above to the visual perception of four-letter words, using the classic dataset of 1180 words employed by McClelland and Rumelhart (1981) in the IAM. Input are b/w images of size 28 × 7 pixels (i.e., patterns over 196 visible units) of words printed with the Siple font. This problem required a DBN with more hidden units: 120 in the first hidden layer and 200 in the second one (see Figure 4).

Two additional examples approach realistic problems: the perception of handwritten digits and visual numerosity perception. The training datasets for these problems contain thousands of samples per category (i.e., digits or numerosity levels) and provide a rich variety of different instances. In the handwritten digit recognition problem, input data consists of 50,000 vectorized gray-level images of size 28 × 28 pixels (i.e., patterns over 784 visible units) that contain handwritten digits from zero to nine (MNIST dataset; LeCun et al., 1998). A robust model of this data would benefit from a hierarchical process that extracts increasingly more complex features (e.g., Gabor filters at the first level, edge detectors in the following layers, etc.). We used the DBN architecture proposed by Hinton and Salakhutdinov (2006) for this task, with three hidden layers of size 500, 500, and 2000 units, respectively. The data of the numerosity perception problem consists of 51,200 vectorized b/w images of size 30 × 30 pixels (i.e., patterns over 900 visible units) that contain up to 32 rectangular objects of variable size. We used the DBN architecture proposed by Stoianov and Zorzi (2012), consisting of two hidden layers of size 80 and 400 units, which was shown to extract abstract numerosity information.

#### Learning parameters

The DBN learning algorithm is governed by few meta parameters. First, the learning rate should be small, typically in the range 0.01–0.1. Second, the use of a momentum coefficient (i.e., a fraction of the previous weight update) is also critical to avoid local minima, and it is usually set to 0.5 at the beginning of training and then increased up to 0.9. Third, network weights should be regularized, that is kept relatively small, by applying a constant weight decrease in the form of a small weight-decay factor of about 0.0001. Finally, weights should be initialized with small random values drawn from a zero-mean Gaussian distribution with standard deviation of 0.01. The initial values of the bias can be set to zero. These and other issues related to training RBMs are discussed in a comprehensive practical guide by Hinton (2010a).

DBNs are trained with the CD learning algorithm, one RBM layer at a time, using as input either the sensory data (first RBM) or the activations of the previous hidden layer (deeper RBMs). This greedy, layer-wise learning procedure can be performed in a completely iterative way, by updating the network weights after each pattern (*on-line* learning). A complete sweep over all training patterns constitutes a learning epoch. In batch (*off-line*) learning, instead, weights updates are computed over the whole training set. A good compromise between these two approaches is to use a *mini-batch* learning scheme, in which the dataset is partitioned into small subsets (i.e., mini-batches) and the weights are updated with the average gradient computed on each subset (Neal and Hinton, 1998). This latter strategy is highly recommended, because it improves the quality of learning by avoiding local minima and it also allows to significantly speed-up the learning phase on multicore parallel implementations (see Testolin et al., 2013, for a mini-batch GPU implementation of deep networks). The mini-batch size should be set between 10 and few hundred patterns.

#### Monitoring learning

The learning progress can be monitored by analyzing the reconstruction error on the training patterns. The mean reconstruction error on the entire training set should fall rapidly at the beginning of learning and then gradually stabilize. However, this measure can be misleading because it is not the objective function optimized by the CD-*n* algorithm, especially for large *n* (Hinton, 2010a). A more precise measure of the performance of the network is to compare the free energy of the training data with that of a sample of held-out patterns (Hinton, 2010a). A final approach to monitor the quality of learning is to regularly perform an additional discriminative task over the learned internal representations, as we will discuss at length below.

#### Sparsity constraints on internal representations

An interesting variant of standard RBMs (and, consequently, DBNs) consists in forcing the network's internal representations to rely on a limited number of active hidden units. In this case the network develops sparse distributed representations, which have many useful properties and appear to be a coding strategy adopted by the brain (Olshausen and Field, 1996; see Olshausen and Field, 2004, for review). Forcing sparseness within a network's hidden layer can be interpreted in terms of inhibitory competition between units (O'Reilly, 2001). A sparse-coding version of the RBM encourages the development of more orthogonal features, which can allow a better pattern discriminability and a more intuitive interpretation of what each unit is representing. In RBMs, sparsity can be obtained by driving the probability *q* of a unit to be active to a certain desired (low) probability *p* (Lee et al., 2008; Nair and Hinton, 2009). For logistic units, this can be practically implemented by first calculating the quantity *q-p*, which is then multiplied by a scaling factor and added to the biases (and, possibly, to each incoming weight) of the hidden units at every weight update. Depending on the number of hidden units, the desired sparsity level (*p*) can be set in the range of 0.01–0.1. Monitoring the distribution of the hidden units activity can be useful to verify that the desired sparsity level is obtained and that the scaling factor is correctly set so that the probability that a unit is active is close to *p* while learning is not hindered (Hinton, 2010a).

### Testing a DBN: read-out of internal representations

When performing a discriminative task, one of the simplest methods is to exploit a linear classifier (e.g., Rosenblatt, 1958), to assign a certain class to each input pattern. The classifier makes a decision by using a linear combination of the input features and this represents its main limitation (Minsky and Papert, 1969). In the case of real sensory signals, this shortcoming is exacerbated by the fact that the feature vectors are high-dimensional and usually lie on highly curved and tangled manifolds (DiCarlo et al., 2012). However, deep belief networks perform a non-linear projection of the feature vector at each hidden layer, gradually building increasingly more complex and abstract representations of the data that eventually make explicit the latent causes of the sensory signal. This hierarchical organization suggests that a linear “read-out” of hidden unit representations should become increasingly more accurate as a function of layer depth. In this perspective, accuracy of linear read-out can be considered as a coarse measure of how well the relevant features are explicitly encoded at a given depth of the hierarchical generative model (see, e.g., Stoianov and Zorzi, 2012; Di Bono and Zorzi, under review). As noted above, linear read-out can also be used to monitor the quality of the representations developed by the deep network during unsupervised generative learning.

The linear read-out on internal representations can be easily implemented using another connectionist module, such as a linear network trained with the delta rule, thereby preserving the biological plausibility of the model. The linear network can also be considered as a response module that supports a particular behavioral task, so that its responses can be assessed against the human data (e.g., numerosity perception in Stoianov and Zorzi, 2012, or location-invariant visual word recognition in Di Bono and Zorzi, under review). For example, Stoianov and Zorzi applied this approach to simulate human behavior in a numerosity comparison task after training a DBN on thousands of images of sets of objects. The internal representations at the deepest layer provided the input to a linear network trained to decide whether the numerosity of the input image was larger or smaller than a reference number. Notably, the responses of this decision module were described by a psychometric function that was virtually identical to that of human adults, with the classic modulation by numerical ratio that is the signature of Weber's law for numbers.

From a practical point of view, delta rule learning can be conveniently replaced by an equivalent method that is computationally more efficient, which relies on the calculation of a pseudo-inverse matrix (Hertz et al., 1991). Formally, data patterns *P* = {*P*_1_, *P*_2_, …, *P*_*n*_} can be associated with desired categories *L* = {*L*_1_, *L*_2_, …, *L*_*n*_} by means of the following linear association:

L=WP

where *P* and *L* are matrices containing *n* column vectors that correspondingly code patterns *P*_*i*_ (sensory data or internal representations) and binary class labels *L*_*i*_, and *W* is the weight matrix of the linear classifier. If an exact solution to this linear system does not exist, a least-mean-square approximation can be found by computing the weight matrix as:

$$W=LP^{+}$$

where *P*^+^ is the Moore-Penrose pseudo-inverse (Albert, 1972)^2^

**Figure 5:**
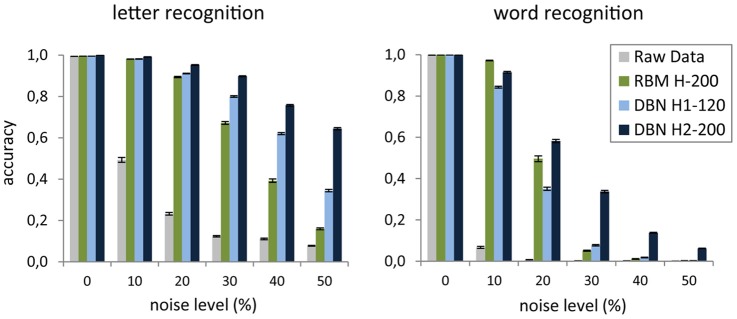
**Mean accuracy of the linear classifier on the task of recognizing each letter of a word (left) and the whole word (right) as a function of noise level applied to the raw images.** Accuracy is averaged over 20 random noise injections and it is computed over the entire dataset of words. Error bars represent SEM. The results are shown for read-out from the two hidden layers of a deep network (DBN), a shallow network (RBM), and raw images.

As an example, we applied the read-out DBN testing method on the internal representations learned for the images of the four-letter words used in McClelland and Rumelhart (1981). We tested two different discriminative problems. The first required the identification of each of the four letters composing a word, using as label a binary vector with one-hot (i.e., localistic) coding of the target letter. The second problem consisted in the identification of the word itself, using as label a binary vector with one-hot coding of the target word. To investigate the quality of the features extracted by deep learning, we compared the classification accuracy on the representations learned at each of the levels of a two-layer DBN (*H*1 = 120 units, *H*2 = 200 units) with that of the representations learned by a single RBM with as many hidden units as the top layer of the DBN (*H* = 200 units). As a baseline, we also measured the classification accuracy obtained by trying to directly categorize the raw input vectors. Note that the read-out of the original data is trivial, due to lack of variability (and noise) in the coding of letters and words (i.e., there is a unique pattern for each letter and word). Indeed, the raw data vectors are linearly separable as shown by the perfect accuracy of the read-out. However, if the input patterns are degraded by adding a certain amount of noise, one should expect a progressive decrease of the classification accuracy when the input representation does not include high-level, invariant features. Indeed, Figure 5 shows that when each word image was corrupted by randomly setting to zero a certain percentage of its pixels, read-out accuracy on the raw pixel data dropped even with a small amount of noise and it approached zero in the word recognition task. As expected, the DBN extracted robust internal representations that were less sensitive to noise. Indeed, both hidden layers supported good discrimination accuracy for letters, whereas only the deepest hidden layer adequately supported word discrimination. Notably, the shallow generative model (RBM) with as many hidden units as the top DBN layer did not unfold word-level information, thereby failing to support robust word recognition (especially for larger noise levels). These results are consistent with the seminal proposal of hierarchical feature processing to yield abstract representations of written words (McClelland and Rumelhart, 1981).

### Analyzing a DBN

#### Discovering learned representations

**Figure 6:**
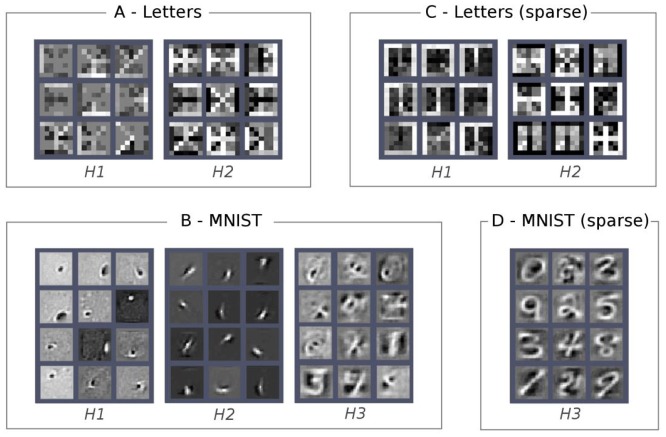
**Visualization of features learned at different hidden layers (*H*_*i*_).** Each square within a layer represents the receptive field of one hidden unit. Excitatory connections are shown in white, whereas inhibitory connections are in black. **(A)** H1 and H2 on single letters (pixelated “Siple font”). **(B)** H1, H2 and H3 on MNIST. **(C)** Sparse H1 and H2 on single letters. **(D)** Sparse H3 on MNIST. From left to right: H1 on single letters (pixelated “Siple font”); H2 on single letters; H1 on MNIST; H2 on MNIST; H3 on MNIST; sparse H1 on single letters; sparse H2 on single letters; sparse H3 on MNIST.

In the previous section we illustrated how it is possible to assess the quality of the internal representations learned at each layer of the hierarchy of a deep belief network by performing a discriminative task. However, this information is tied to a given classification task and is therefore limited in scope. Moreover, the supervised classifier operates on the pattern of activity over an entire hidden layer, that is a distributed representation encoding a variety of micro-features (Hinton et al., 1986) representing task-independent statistical regularities of the data. A very simple but informative approach to investigate the role of a particular unit in the network consists of visualizing its connection weights using the original structure of the data (e.g., the 2D image in our visual perception examples). This is particularly intuitive for the first hidden layer, where the weight matrix defines how the visible units contribute to the activation of each hidden unit. We can therefore visualize the “receptive field” of each hidden unit by plotting the strength of its visible-to-hidden connections. The same principle can be applied to the deeper layers of the DBN, by combining their weight matrix with those of the lower layers. A straightforward way is to use a linear combination of the weight matrices, possibly imposing a threshold on the absolute values of the weights in order to select only strong connections. This allows to visualize the receptive field learned at a layer *k* as a weighted linear combination of the receptive fields learned at level *k-1* (Lee et al., 2008, 2009). The main drawbacks of this technique are that one has to manually choose threshold values and that non-linearities between layers are not considered, with the risk of losing relevant information. Nevertheless, this method can provide good visualization of the learned features even without imposing a threshold on the weights (see Figure 6).

Using the above method, we analyzed the receptive fields of the hidden units of DBNs trained on images of letters as well on the handwritten digits of the MNIST dataset. In the letter perception task, we found that most of the units of the first hidden layer were tuned to basic geometric features, whereas most of the units of the second hidden layer were tuned to a composition of these features (see examples in Figure 6A). The greater image resolution and variability of the handwritten digits pose a much more complex visual problem, which induced the emergence of a more structured hierarchy of features in the DBN. As shown in Figure 6B, the first hidden layer learned simple and localized visual features (mostly Gaussian and Gabor filters), resembling those found in the primary visual cortex. The second hidden layer combined these features into edges, lines, and strokes detectors. Finally, the third hidden layer extracted even more complex visual features that resemble parts of digits. Note that the finding of low-level visual features (basis functions) in the first hidden layer is common to many problems that involve a large variability in the training images (see, e.g., Lee et al., 2008; Stoianov and Zorzi, 2012).

Applying sparsity constraints on the internal representations further improves the quality of the emerging features. For example, a sparse DBN trained on patches of natural images developed complex receptive fields (e.g., T-junctions) in the second hidden layer that were very similar to those found in area V2 of the visual cortex (Lee et al., 2008). Our sparse DBN simulations also resulted in an increase of the complexity of the emergent features. For example, the letter perception network encoded more letter-like features in the second hidden layer (Figure 6C) and the handwritten digit perception network learned shape-specific detectors in the third hidden layer (Figure 6D).

A more sophisticated approach to investigate the features encoded by a hidden unit is to find its preferred input stimuli, as done by neurophysiologists in single-cell recording studies. The basic idea is to probe the network on a variety of input patterns, each time recording the neural response and then looking for possible regularities. This approach can be very effective if we have an idea about which type of patterns are more likely to elicit specific responses (for example, responses to bigrams after training on words; Di Bono and Zorzi, under review). However, if we cannot make assumptions about the nature of the preferred stimuli, this method becomes computationally intractable because it would require testing the network on an exponential number of possible input patterns. Nevertheless, this problem can be solved by formulating it as an *optimization problem*, where the goal is to find the input pattern that maximizes the activation of a certain hidden unit given the processing constraints imposed by the network (Erhan et al., 2009). Formally, if θ denotes the deep network parameters (weights and biases) and *h*_*ij*_(θ, *x*) is the activation of a given unit *i* from a given layer *j* in the network, then *h*_*ij*_ is a function of both θ and the input sample *x*. Assuming that the vector *x* has a bounded norm and after learning the parameters are fixed, then the problem of maximizing the unit activation is:

$$x^{*}=\underset{x}{\arg\max}\;h_{ij}(\theta ,\;x)$$

Although this is a non-convex optimization problem, it has been empirically shown that good local minima can be found (Erhan et al., 2009). This method has been recently used to investigate whether high-level, class-specific feature detectors can emerge in very-large-scale deep unsupervised learning (i.e., using millions of images for training; Le et al., 2012). The impressive result was that it is indeed possible to learn highly complex and abstract features at the deepest layers, such as prototypical faces (Le et al., 2012).

A different approach can be used if we expect monotonic response of some hidden units to a given property of the data. The individuation of these detectors is based on regressing the property of interest (or even multiple properties) onto the response of each hidden unit. A high absolute value of the normalized regression coefficient indicates sensitivity of the hidden unit to the property of interest; this might also indicate selectivity when combined with small (near-zero) regression coefficients for other properties. Using this method, Stoianov and Zorzi (2012) discovered detectors in the second hidden layer of their DBN tuned to visual numerosity but insensitive to other important visual properties like cumulative area. Di Bono and Zorzi (under review) also used this method to investigate word selectivity in their DBN model of visual word recognition. After finding the preferred word for a given hidden unit, its word selectivity was assessed by recording the response to all other training words and performing a regression analysis using the orthographic (i.e., Levenshtein) distance from the preferred word as predictor.

#### Sampling from the generative model

**Figure 7:**
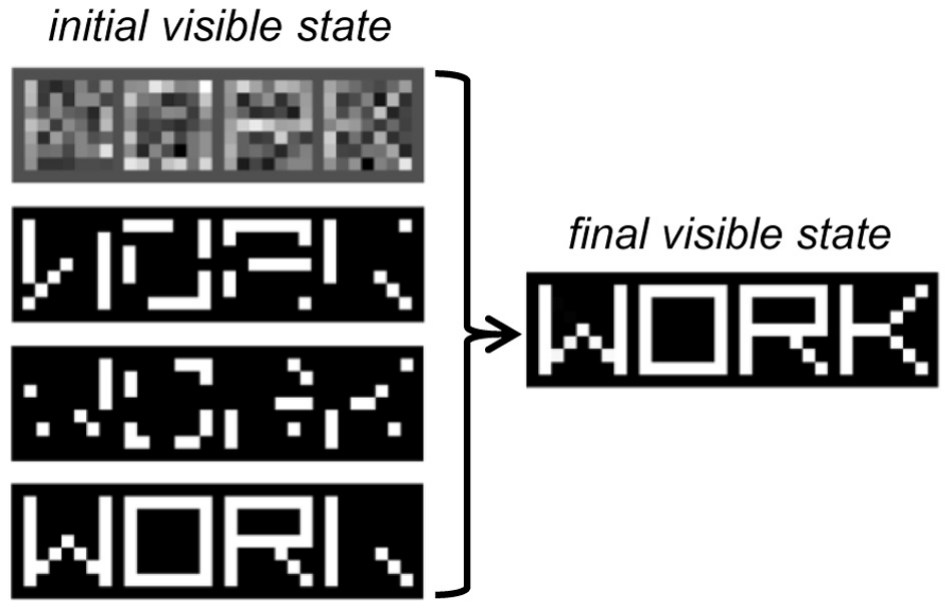
**Inference in the word perception DBN when the word image “WORK” is presented as input under different types of noise.** From top to bottom: Gaussian noise, binary noise (30%), binary noise, (50%), occlusion noise. The final state of the visible units, identical across the four noise conditions, is shown on the right.

Up to this point, we only discussed methods that investigate the *bottom–up* processing of sensory data. However, a deep belief network is a *generative* model, and it can be very useful to assess the *top–down* generation of sensory data, as well as the mixing of bottom–up and top–down signals during inference in a noisy situation. In one scenario, we can provide to the model a noisy input pattern (e.g., randomly corrupted or partially occluded) and let the network find the most likely interpretation of the data under the generative model. This process requires the iteratively sampling of the states of the network until an equilibrium activation state is reached, which in DBNs can be efficiently done using block Gibbs sampling (see Box 3). As an example, in Figure 7 we show the result of inference in the word perception DBN when four different noisy versions of the same image are given as input to the model. Note that the visible units settle onto an activation state corresponding to the correct word image.

**Figure 8:**
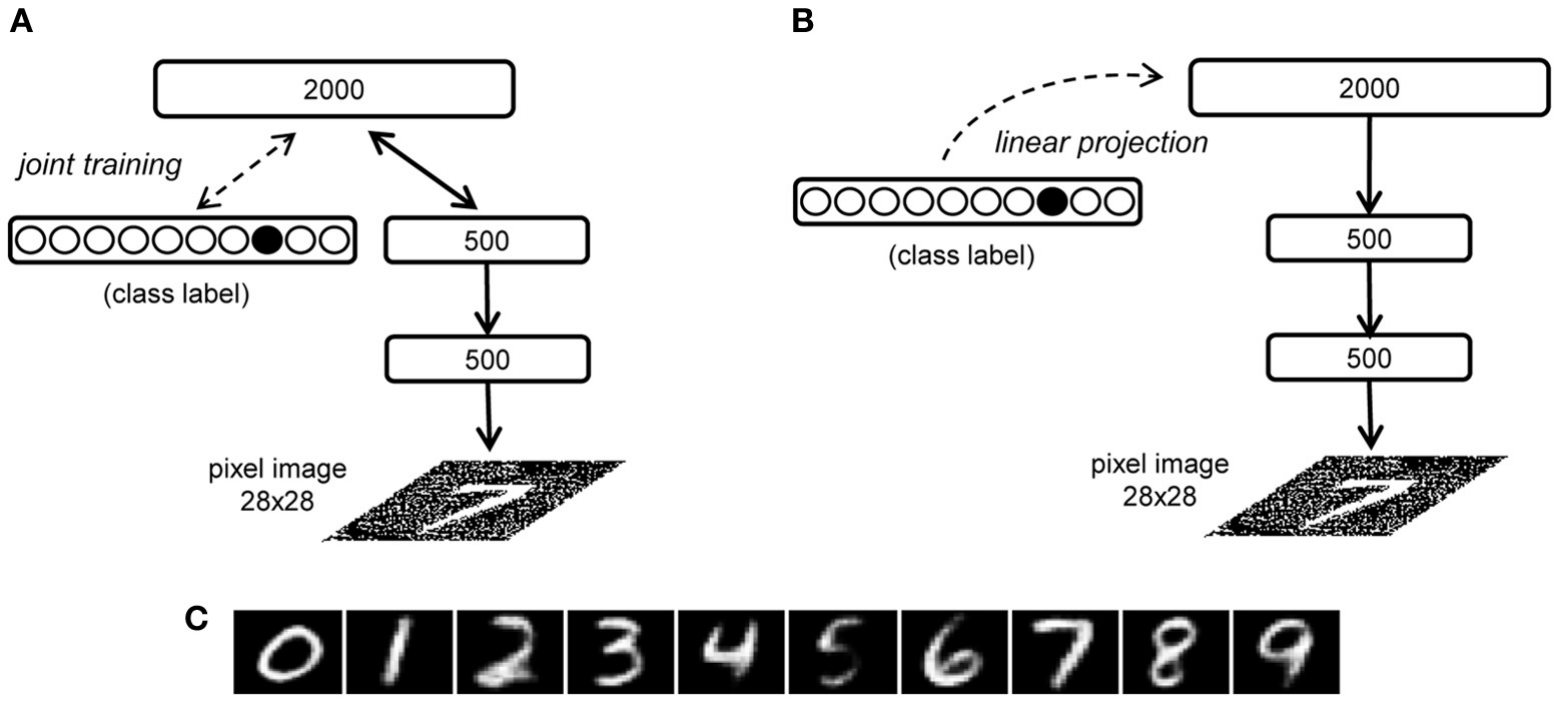
**Illustration of the prototype generation methods in the handwritten digit recognition model. (A)** The RBM involving the third hidden layer is jointly trained on the internal representation of the second hidden layer and an additional set of units representing the digit classes (Hinton et al., 2006). **(B)** Our linear projection method: class label units are only added after the complete DBN training and are associated to the third hidden layer representations by means of a linear mapping. **(C)** Digit prototypes generated using the linear projection method.

We can also study the generative capability of a DBN when the visible units are not clamped to an initial state, and the network is therefore let free to autonomously produce a sensory pattern through a completely top–down process. This generative process can be constrained to produce “class prototypes” by adding a multimodal RBM on the top of the network hierarchy (Hinton et al., 2006), which is jointly trained using two input sources, one containing the internal representation learned by the DBN and the other encoding the corresponding label. For example, in the handwritten digit recognition model, input to the multimodal RBM is provided by the second hidden layer (500 units) and by 10 units representing the image label (one unit for each possible digit class) (see Figure 8A). After learning, the label units can be clamped to a certain state (e.g., with only the unit corresponding to the class “7” active) and the top RBM settles to equilibrium, thereby recovering the internal representation of the given digit class. The generative connections of the DBN can then be used to obtain an image on the visible layer in a single top–down pass. The image generated can be thought of as the model's prototype for the corresponding abstract representation.

Here we propose an interesting, more simple variant of the top–down generation of the learned prototypes. Instead of jointly training the top-level RBM using the internal representation of images and the corresponding class label, and then performing Gibbs sampling until equilibrium with the label units clamped to a certain class, we can try to directly map the class label and the internal representation through a linear projection (see Figure 8B). This mapping is analogous to the read-out module previously discussed but it works in the opposite direction. Prototype generation can thus be performed by associating the class vectors *L* with the internal representations *P* learned by the DBN through a weight matrix *W*_2_:

$$P=W_{2}L$$

$$W_{2}=PL^{+}$$

As in Hinton et al. (2006), after computing the internal state *P* at the deepest layer, a single top–down pass through the generative connections of the DBN produces the prototype for the specific class. Figure 8C shows the prototypes generated for each digit class of the MNIST dataset using this linear projection method. Note that this method can be readily extended to more complex scenarios that involve a mapping between internal representations learned by different networks (which may reflect knowledge about different domains or sensory modalities).

Finally, it is worth noting that the quality of inference when sampling from the generative model can be improved if the single top–down pass is replaced by an interactive process, as proposed in a recent variant of the DBN known as Deep Boltzmann Machine (Salakhutdinov and Hinton, 2009).

## Discussion

Understanding how cognition and language might emerge from neural computation is certainly one of the most exciting frontiers in cognitive neuroscience. In this tutorial overview we discussed a recent step forward in connectionist modeling, which allows the emergence of hierarchical representations in a deep neural network learning a generative model of the sensory data. We started by reviewing the theoretical foundations of deep learning, which rely on the framework of probabilistic graphical models to derive efficient inference and learning algorithms over hierarchically organized energy-based models. We then provided a step-by-step tutorial on how to practically perform a complete deep learning simulation, covering the main aspects related to the training, testing and analysis of deep belief networks. In our presentation we focused on examples that require the progressive extraction of abstract representations from sensory data and that are therefore representative of a wide range of cognitive processes. In particular, we showed how deep learning can be applied to the classic letter and word perception problem of McClelland and Rumelhart (1981). In addition to providing a useful toy example of modeling based on deep learning, the emergent properties of the model revisit key aspects of the seminal IAM and suggest a very promising research direction for developing a full-blown deep learning model of visual word recognition. Indeed, up-scaling the present toy model is likely to be successful because deep learning is particularly suited to capture features hierarchies over large training datasets with great pattern variability. This aspect was present in two additional problems that complemented our tutorial with more realistic simulations, that is, handwritten digit recognition (LeCun et al., 1998) and visual numerosity perception (Stoianov and Zorzi, 2012). Together, the various simulations illustrate the strength of the deep learning approach to cognitive modeling.

Deep unsupervised learning extracts increasingly more abstract representations of the world, with the important consequence that explanatory factors behind the sensory data can be shared across tasks. The hierarchical architecture captures higher order structure of input data that might be invisible at the lower levels and it efficiently exploits features re-use. The idea that learned internal representations at the deepest layers can be easily “read-out” is consistent with the notion of “explicitness of information” articulated by Kirsh (1990), who argued that explicitness is tightly related to the processing system which uses it. Within this perspective, the degree of explicitness is better linked to the usability of information rather than to its form (i.e., how quickly it can be accessed, retrieved or in some other manner put to use). This idea has been further extended by Clark (1992), who proposed to take into account also the multi-track usability of stored information: “Truly explicit items of information should be usable in a wide variety of ways, that is, not restricted to use in a single task” (p. 198). Note that this conception of abstract representations that can be shared across tasks or even across domains is particularly useful in the context of modeling language processing.

Efficient generative learning in neural networks is a recent breakthrough in machine learning and its potential has yet to be fully unfolded. In particular, the extension of RBMs to the temporal domain (Sutskever et al., 2008; Taylor and Hinton, 2007) is a very promising avenue for research. Indeed, generative networks that learn the temporal dynamics of the data could anticipate relevant events in the environment, using the history of the system as context to make accurate predictions about the incoming information, as proposed by the predictive coding framework (Huang and Rao, 2011; Clark, 2013). Learning and processing of sequential information is also a key aspect of cognition and it is particularly ubiquitous in language processing (Elman, 1990). An initial exploration of this direction is the use of the Recurrent Temporal RBM (Sutskever et al., 2008) for learning orthographic structure from letter sequences (Testolin et al., 2012, submitted).

It is worth noting that deep generative network models of cognition can offer a unified theoretical framework that encompasses classic connectionism and the structured Bayesian approach to cognition. Structured Bayesian models of cognition (for reviews see Chater et al., 2006; Griffiths et al., 2010) assume that human learning and inference approximately follow the principles of Bayesian probabilistic inference and they have been used in the last few years to address a number of issues in cognitive science, including language processing (Chater and Manning, 2006, for review). However, Bayesian models are typically formulated at the level of “computational theory” (Marr, 1982) rather than at the process level that characterizes other cognitive modeling paradigms like connectionism (for further discussion see McClelland et al., 2010; Jones and Love, 2011). This implies limits on the phenomena that can be studied with the Bayesian approach, because only problems of inductive inference or that contain an inductive component are naturally expressed in Bayesian terms (Griffiths et al., 2008). In contrast, computational models of cognition based on deep neural networks and generative learning implement the probabilistic approach in a neural-like architecture and can provide an emergentist explanation of structured representations that is in line with the connectionist tradition (McClelland et al., 2010). Their probabilistic formulation not only allows to deal with ambiguity of sensory input and with the intrinsic uncertainty of environmental dynamics, but it also provides a coherent theory about how learning can integrate new evidence to refine beliefs of the model. Importantly, there is no need to have an external signal that guides learning, because the aim is to reproduce incoming information as accurately as possible by discovering its hidden causes (that is, learning can be seen as a stochastic inference problem).

In conclusion, we believe that the focus on deep architectures and generative learning represents a crucial step forward for the connectionist modeling enterprise, because it offers a more plausible model of cortical learning as well as way to bridge the gap between emergentist connectionist models and structured Bayesian models of cognition.

### Conflict of interest statement

The authors declare that the research was conducted in the absence of any commercial or financial relationships that could be construed as a potential conflict of interest.

## Footnotes

^1^A variety of multicore implementations (MATLAB and Python on graphic cards; Octave/MPI on a multi-core cluster) is described in Testolin et al. (2013) and the source codes can be found at: http://ccnl.psy.unipd.it/research/deeplearning

^2^In some high-level programming languages, this operation is readily available. For example, in MATLAB/Octave we can use the *pinv()* function, *W* = *L*^*^*pinv*(*P*), or the left matrix divide (a.k.a. “backslash”) operator, *W* = (*P*′\*L*')'.
